# Supplementary material for: Evaluation of Sex Differences in Murine Diabetic Ketoacidosis and Neutropenic Models of Invasive Mucormycosis
Source: J Fungi (Basel). 2021 Apr 18;7(4):313. doi: 10.3390/jof7040313 (PMC8072604; doi:10.3390/jof7040313)
Supplement: Supplementary file 1 [file jof-07-00313-s001.zip › jof-1195257-supplementary.pdf]

Figure S1

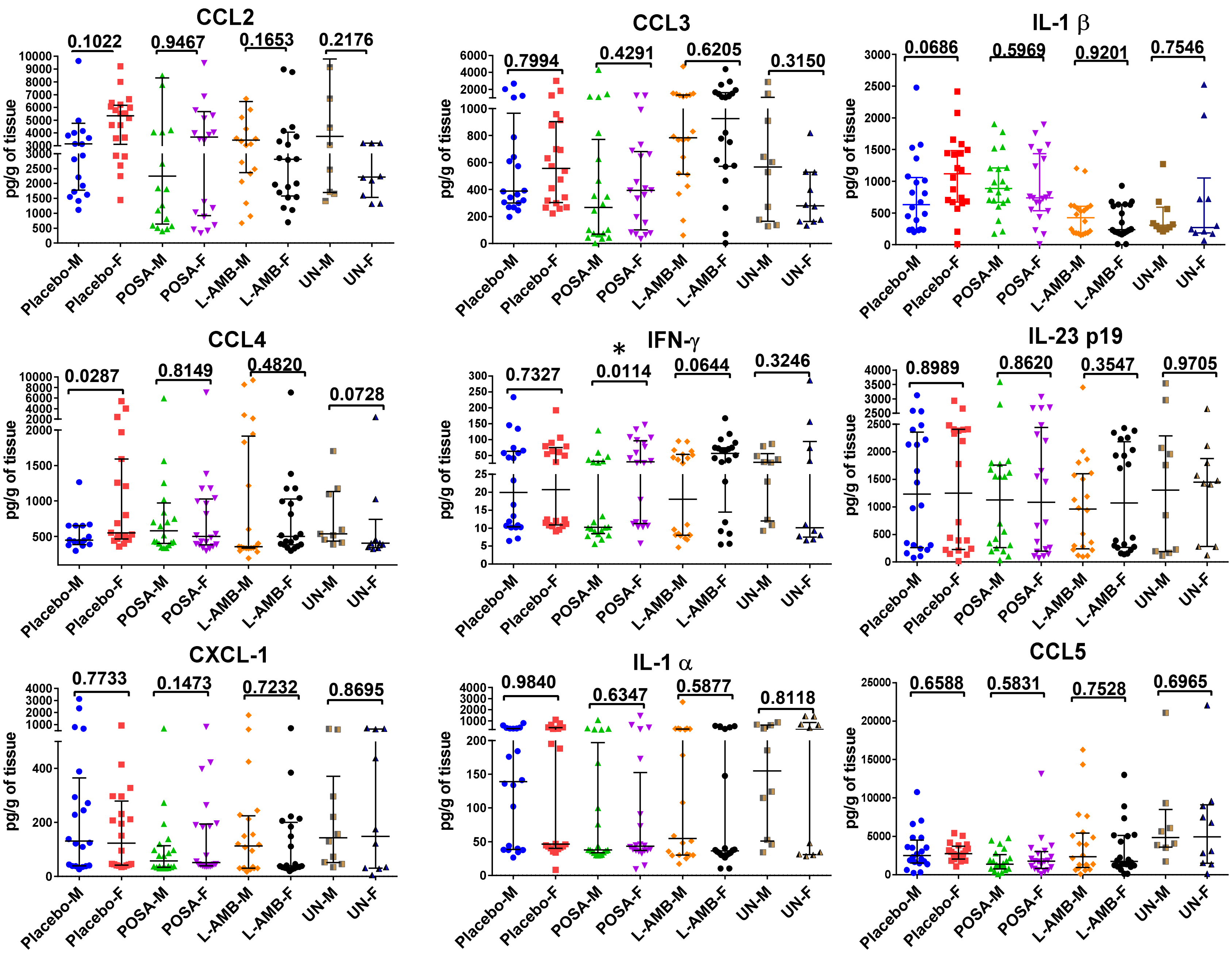
CCL2
0.1022
0.9467
0.1653
0.2176
CCL3
0.7994
0.4291
0.6205
0.3150
IL-1 β
0.0686
0.5969
0.9201
0.7546
CCL4
0.0287
0.8149
0.4820
0.0728
IFN-γ
*
0.7327
0.0114
0.0644
0.3246
IL-23 p19
0.8989
0.8620
0.3547
0.9705
CXCL-1
0.7733
0.1473
0.7232
0.8695
IL-1 α
0.9840
0.6347
0.5877
0.8118
CCL5
0.6588
0.5831
0.7528
0.6965
pg/g of tissue
Placebo-M
Placebo-F
POSA-M
POSA-F
L-AMB-M
L-AMB-F
UN-M
UN-F

Figure S1

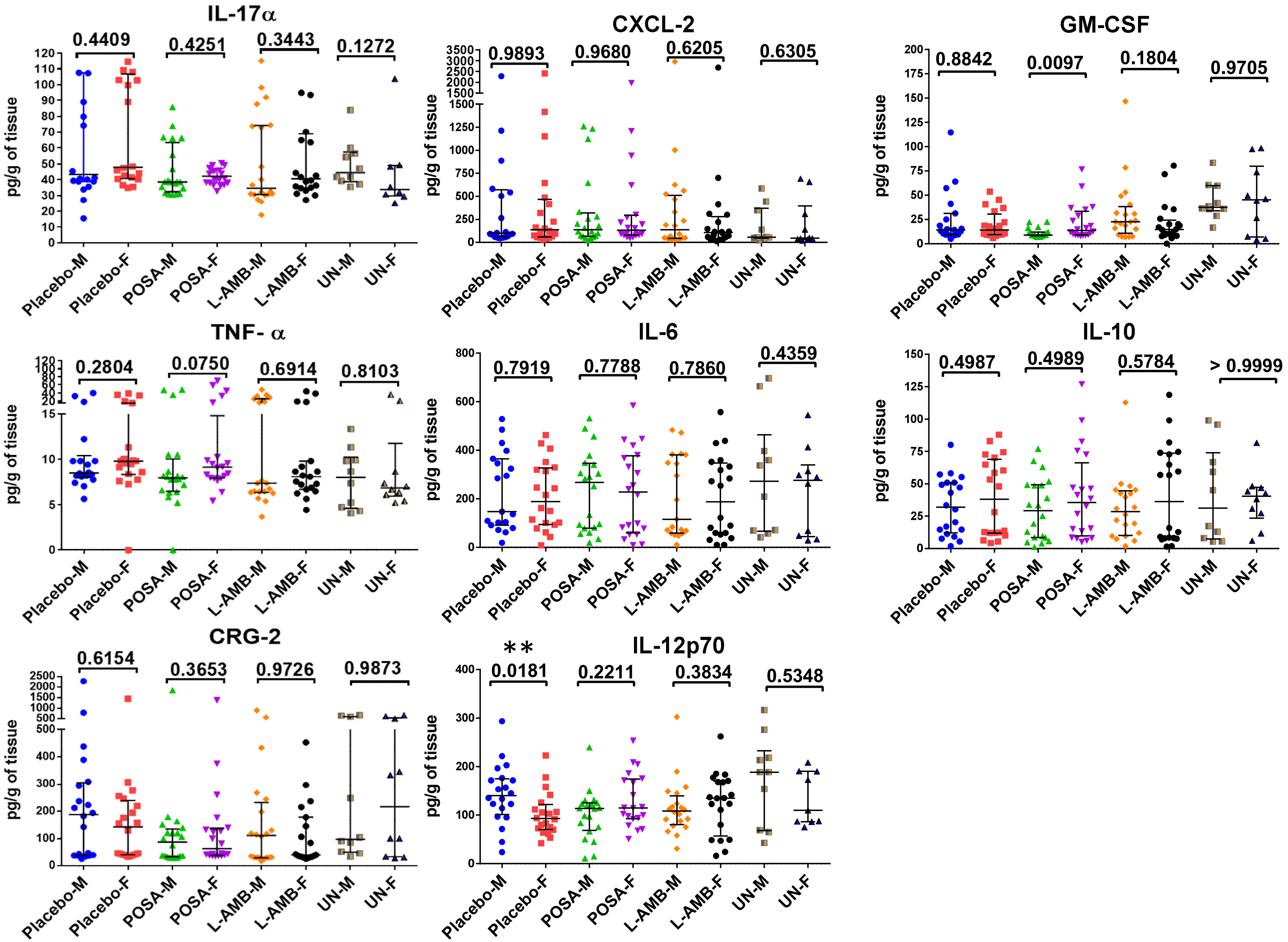

Figure S1. Cytokine analysis of lung homogenate from female or male DKA mice infected with *R. delemar.* Lung homogenate cytokines (n=20 from two independent experiments) were analyzed by luminex. *P = 0.01 for interferon-γ of POSA F *vs.* POSA M. **P = 0.02 for IL-12p70 of Placebo F *vs.* Placebo M.

Figure S2

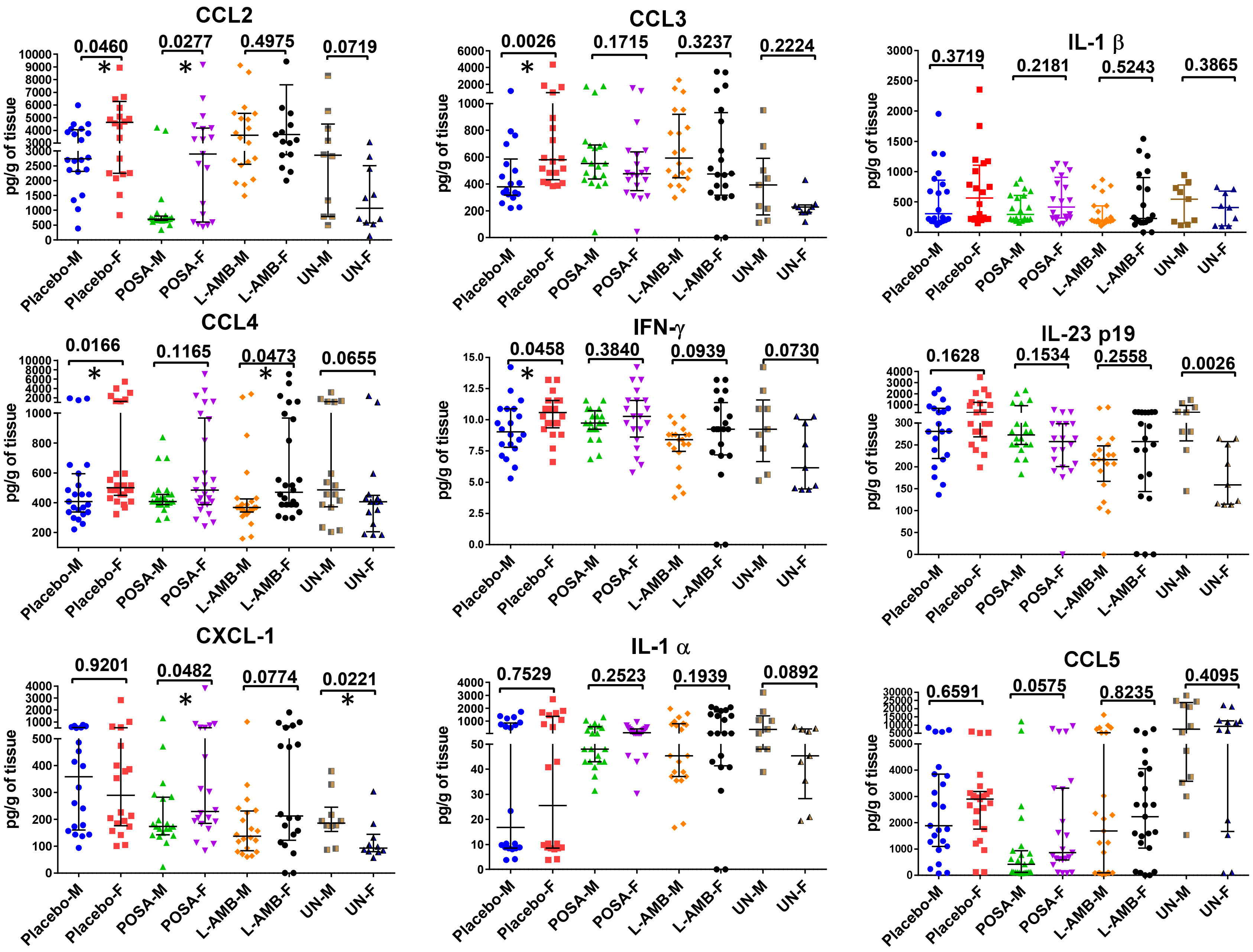
CCL2
0.0460
0.0277
0.4975
0.0719
CCL3
0.0026
0.1715
0.3237
0.2224
IL-1 β
0.3719
0.2181
0.5243
0.3865
CCL4
0.0166
0.1165
0.0473
0.0655
IFN-γ
0.0458
0.3840
0.0939
0.0730
IL-23 p19
0.1628
0.1534
0.2558
0.0026
CXCL-1
0.9201
0.0482
0.0774
0.0221
IL-1 α
0.7529
0.2523
0.1939
0.0892
CCL5
0.6591
0.0575
0.8235
0.4095
pg/g of tissue
Placebo-M
Placebo-F
POSA-M
POSA-F
L-AMB-M
L-AMB-F
UN-M
UN-F

Figure S2

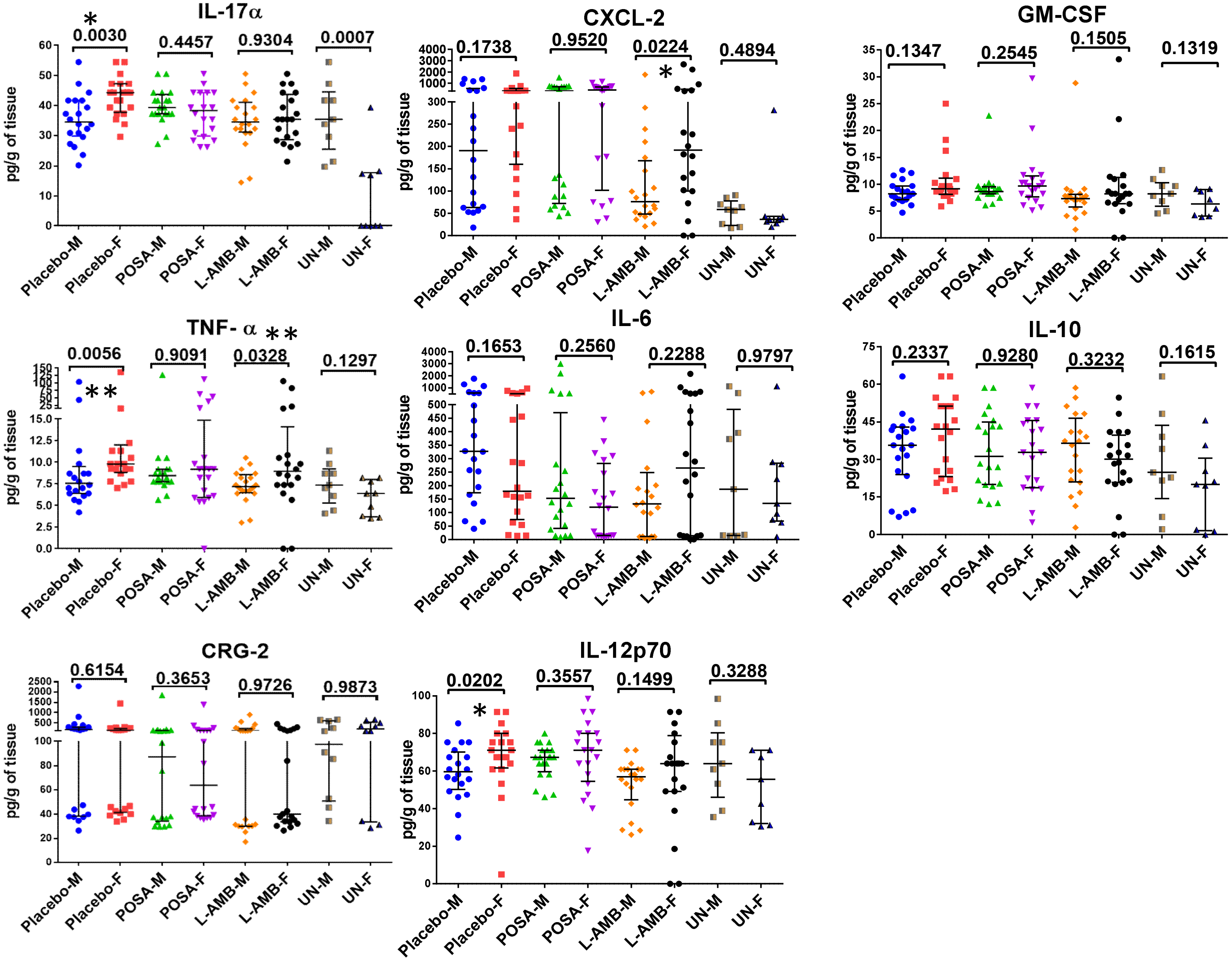

Figure S2. Cytokine analysis of lung homogenate from female or male DKA mice infected with *M. circinelloides f. jenssenii.* Lung homogenate cytokines (n=20 from two independent experiments) were analyzed by luminex. **P* < 0.005 for IL-17α of Placebo F *vs.* Placebo M. ***P* < 0.05 for TNF-α of L-AMB F *vs.* L-AMB M.

Figure S3

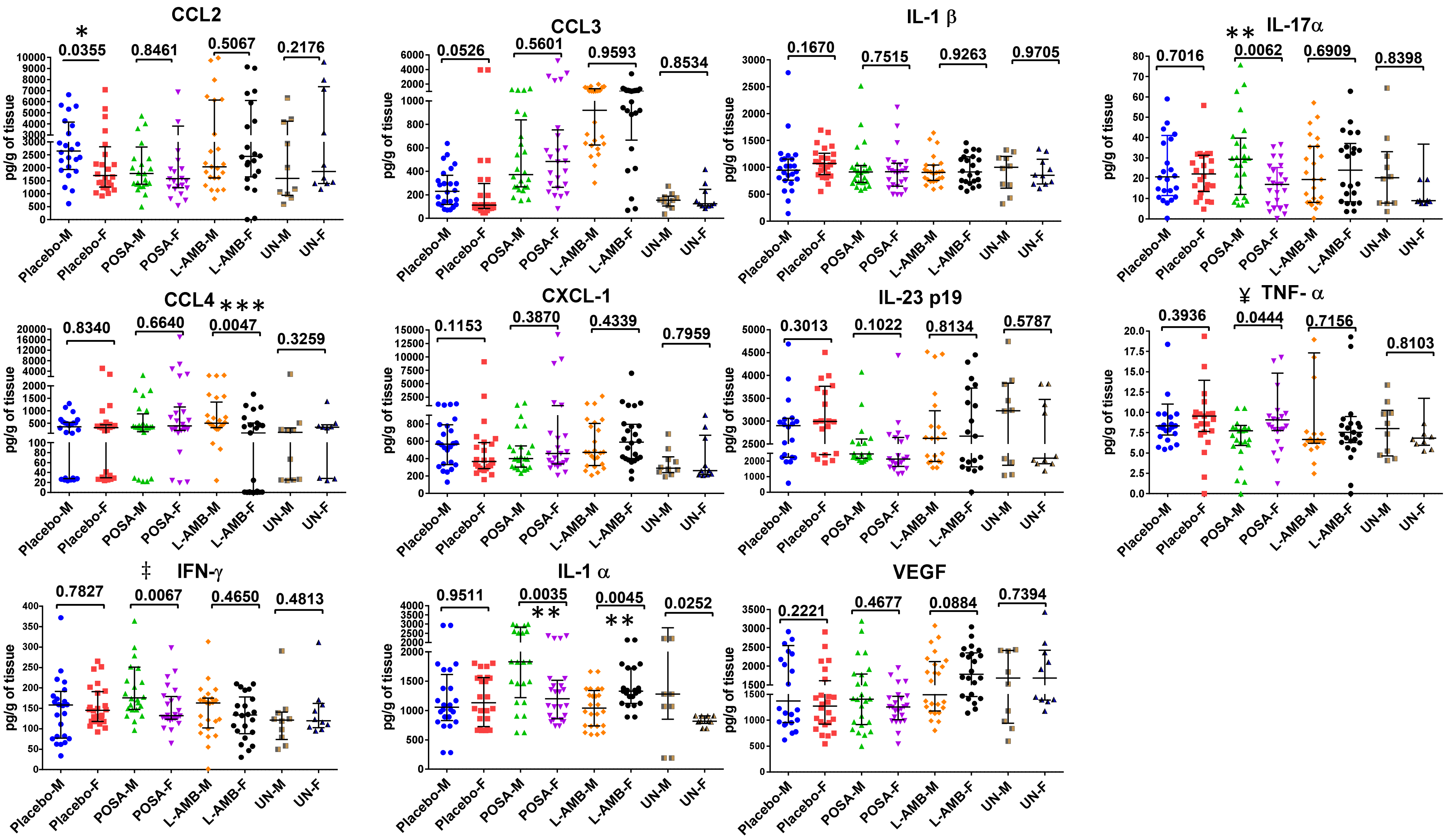

Figure S3. Cytokine analysis of lung homogenate from female or male neutropenic mice infected with *M. circinelloides f. jenssenii.* Lung homogenates cytokines (n=23 from two independent experiments) were analyzed by luminex. *$P < 0.05$ for CCL2 of Placebo F *vs.* Placebo M. **$P < 0.001$ for IL-17α of POSA F vs. POSA M. ***$P < 0.005$ for CCL4 of L-AMB F *vs.* L-AMB M. ¥$P < 0.05$ for POSA F *vs.* POSA M. ‡$P < 0.01$ for POSA F *vs.* POSA M.
